# American Dental Society of Europe

**Published:** 1886-01

**Authors:** 


### AMERICAN DENTAL SOCIETY OF EUROPE.

### THIRTEENTH ANNUAL MEETING, HELD IN BERLIN, AUGUST, 1885.

REPORTED EXPRESSLY FOR THE INDEPENDENT PRACTITIONER.

(Continued from page 655, Vol. VI.)

Dr. Miller delivered an address, accompanied by illustrations, on some phenomena connected with caries of the human teeth and their explanation. The following is a brief summary: The speaker asked indulgence of the society in presenting so repeatedly a subject of this nature, but having started out on a certain path he thought it best to follow it to the end. He compared the human mouth to a wilderness, in which such a variety of agents and conditions are present that, until they are separated and the action of each one determined individually, it is an utter impossibility to come to a safe and certain conclusion as to which of them may be evil, and the active agents in the production of the various diseases met with in the oral cavity. He referred more particularly to the numerous fungi in the oral cavity, of which he himself had isolated no less than twenty-five different kinds.

The method of obtaining pure cultures was clearly explained by the following simile: "Suppose we have a hand full of seeds of various kinds — wheat, rye, barley, oats, corn, etc., etc — and that the separate grains are invisible to the naked eye. How can we separate these different seeds so as to obtain a number of piles which shall contain seed of one kind only? We should go with the grains to a ploughed field and sow them, and when they have sprung up and brought forth fruit we would gather the heads of wheat into one bundle, the heads of rye into another, and so on, and in this way obtain a number of bundles, each containing grain of one kind only. Now, if we have a mixture containing any number of different micro-organisms, we may effect the separation in exactly the same manner, except that we sow the fungi on nutritive gelatine instead of ploughed ground."

Prof. Miller then demonstrated the method of sowing the fungi in gelatine, and of gathering the different kinds which spring up, into separate tubes, as described in this journal. He called particular attention to one fungus commonly found in the human mouth, which produced large quantities of gas, the gelatine being completely torn to pieces by the same.

He had also frequently met with this organism in the intestines, where it may give rise to the well known disorders accompanying development of gas in the alimentary tract.

He then described a number of experiments made with glass bulbs, representing the crown and root of a tooth. These had been filled twenty-four hours previously with nutrient material, infected with a bacterium which had been found in a gangrenous pulp, and the apices closed. In breaking the points a portion of the contents of the bulb was forced out by the pressure of the gas which had accumulated in the bulb. He called attention to the fact that an attack of pericementitis with severe pain appears sometimes without any previous warning, and thought that the experiment furnished an explanation of such cases, the gas gradually accumulating in the root until the pressure becomes sufficiently great to force through portions of the pulp by which the apex may have been closed. Potato cultures of a large number of chromogeneous micro-organisms were shown, including *Micrococcus prodigiosus*, *Bacillus ruber*, *Saccharomyces glutinis*, etc., etc., also three green and two yellow fungi from the human mouth. He considered it a great mistake to look upon any of these color-producing fungi as the direct cause of the different colors of the carious dentine. These, leaving out of account the discoloration produced by tobacco, etc., Prof. Miller accounts for in an altogether different manner. He illustrated his view of the subject with five tubes, originally containing absolutely colorless transparent culture gelatine. These had all been inoculated at different times with the same colorless fungus.

In the first tube, three weeks old, the gelatine was partially liquified, but still colorless. In the second, five weeks old, the gelatine was completely liquified and of a yellowish brown tinge. In the third tube, seven weeks old, the color was somewhat darker. In the fourth, nine weeks old, it was dark brown; and in the fifth, six months old, the liquid was completely evaporated, leaving a black residue, in color identical with the so-called *caries nigra*, which we see on approximal surfaces where caries had once begun, and later, generally through the extraction of the neighboring tooth, had ceased. There we had nearly every variety of color seen in carious dentine, resulting from the inoculation of a colorless medium by a colorless organism. Cultures of very many micro-organisms, especially of such as liquify the gelatine, become yellowish brown or dark brown in the course of a few weeks. The discoloration is no doubt chiefly due to the action of the products of decomposition upon the culture material.

The following paper was read by Dr. Sachs:

THE HERBST METHOD.

Any improvement which tends to facilitate the operation of filling teeth should be gratefully accepted by every dentist, for probably no dental work is connected with so many difficulties, demands so much trouble, perseverance, patience, skill and practice, as filling teeth with gold.

It is now some six years since Mr. Herbst brought his method to the notice of the profession, but, although he spared no trouble to make it generally known, it does not, as yet, seem to have been adopted by many. Even though one occasionally reads in the journals of successes achieved by some of the followers of Mr. Herbst, I must maintain that the value of this method is at present of a highly problematical character.

I base this assertion on an experience of eighteen months. I have spared neither time, gold nor trouble, in order to test the invention most conscientiously, and think I have to-day, after a year and a half, experience enough to pronounce judgment upon it. The principal advantages claimed by Mr. Herbst for his method are:

1. It enables one to operate two or three times faster, while the result equals the finest work insured by any other method.

2. It shortens, in proportion, the pain of operating for the patient.

3. It saves diseased teeth from the strokes of the mallet, and makes filling the cavity nearly or quite painless.

4. It saves us dentists more than half the time, and trying labor.

All this sounds very well and tempting, but unfortunately the conclusion appears inevitable that the above assertions are, for most dentists to say the least, not without error. Though Mr. Herbst is able to make a gold filling with great rapidity by his method, that does not prove that a practiced operator may not, in the same time, fill the same cavity by hand pressure just as well, or even better. Furthermore, I assert that it is impossible to make a large gold filling with the rotation method alone of which the surface will not be defective.

The specimens mounted in plaster, which were sent to the various meetings, cannot be conclusive to an experienced operator, though they may take the fancy of a beginner.

The advantage claimed in No. 3 is only apparent, since teeth with diseased periosteum, which alone can be meant, should not be filled with gold by any method until the diseased condition has been removed. The rotation method always requires a certain pressure of the instrument in order to condense the gold sufficiently. This pressure, in connection with the friction, frequently generates so much heat that severe pain results to the patient, so that this advantage does not in reality exist; and if Mr. Herbst declares that filling can henceforth be effected with hardly any pain, I remark that not the filling, but the excavating and preparing of the cavity is by far the more painful part of the operation, and against this evil his invention is as powerless as the old systems.

As an especial disadvantage of this method, I must mention the preparation of the cavity necessary in many cases. To suit his method Mr. Herbst often sacrifices sound, useful tooth-substance, which I should by all means try to preserve, for we have not the moral right to mutilate a tooth simply for the sake of making a large, conspicuous gold filling. It is also a great disadvantage that Mr. Herbst nearly always tries to fill approximal cavities of incisors and cuspids from the labial surface, because he is thereby often forced to destroy part of the labial wall, which might be preserved if the fillings were made by other methods.

The advocate of the German method in America, Dr. Bödecker, teaches that it is advisable to condense the surface of the filling with the mallet. Why is this necessary if the rotation method is capable of producing a hard, compact gold filling? Rotation by means of the burring engine alone, seems not to have satisfied even the inventor, for he also uses hand instruments for condensing the gold in the cavity. That these instruments are not furnished with serrations, but have smooth points, only roughened on sandpaper, is nothing new. The vaunted rapidity is often counterbalanced by the fact that most cavities must be changed to so-called central cavities, by means of shellac, pins and other auxiliaries, a preparation which in many cases would require almost as much time as the filling itself. As already stated, I have practiced the Herbst method for eighteen months with great care and attention, as the advantages claimed for it by the inventor certainly sound tempting enough to induce any one to try it for the sake of time and trouble saved, but I could not succeed in producing work which could meet the test which I am accustomed to require of a good filling, neither was I able to save any time.

I have, therefore, reason to take for granted that it is not possible, by the rotation method, to make a faultless gold filling in one-half or one-third the time required by other methods, and that, too, with considerably less inconvenience to the patient. I, as a German, of course heartily welcome any German invention with just pride, but when one gives it the name of "German method" in contradistinction to American method, it can only humiliate us if it is found out that it neither surpasses nor even equals the methods used heretofore. I think it advisable, therefore, to call the invention the "Herbst Method," all the more as it is the custom to call novelties by the name of their inventor. It is also only just to Mr. Herbst that his priority should be recognized in the name of the method, and just to German dentists that he, and not they, should assume the responsibility of the new method.

*Dr. Patton*—I have had an opportunity to see Mr. Herbst demonstrate his method of rotatory filling, and must say that it failed to impress me in any way. I could not see the advantage to be gained by it. He cuts away large portions of enamel, weakening the teeth in order to get room to work his rotatory instruments. He picks up his gold with a hand-plugger and presses it into place, then applies the rotatory pressure, burnishing the surface of the gold, and, probably, by the friction of rapid revolutions, causing an adhesiveness. On examination of what I saw, however, the filling was not evenly condensed, and if the last layer were removed it could be pulled to pieces in flakes, which shows that the gold was not really thoroughly condensed.

Now I work altogether with hand pressure, and I fail to see how I can gain time or make a more rapid operation by the new method. I can condense with the hand in the time I take to change instruments, besides getting my pluggers into positions and corners that he cannot reach, and which he himself, when it comes to this, fills up with a hand plugger. I fail to appreciate the method.

*Dr. Richter*—I have used the Herbst method very considerably, and, having made a large number of fillings in front teeth, I was obliged to remove all of them that did not fall out, on account of imperfections. I sometimes use the method, however, for fillings on the grinding surface of molars, where it works very rapidly.

*Dr. Kirschner*—Expressed similar views.

*Dr. Cohn*—Recommended, for rapid work, rolled gold wrapped in No. 4 foil.

*Dr. Miller*—Being asked for his experience, said: I have been very anxious to find out, if possible, the real value of the Herbst method, feeling it my duty, if the method is a superior one, to introduce it here at the Dental Institute. I was favorably impressed by the results obtained in an examination of a test filling made out of the mouth, but though I had made a few fillings under the eye of the inventor, I had not had sufficient experience in the use of the method to form an opinion as to its utility. I accordingly addressed letters of inquiry to a considerable number of dentists, asking for their experience in the use of the Herbst method. The answers which I received were, on the whole, unfavorable. Some had not used the method, but had seen beautiful work done by it. Some could not see any advantage to be gained by it. Some pronounced it utterly worthless. On the other hand, we have the opinion of Dr. Bödecker, always entitled to the greatest respect, who enthusiastically endorses it. For my own part, I am not able at present to come to a definite conclusion, but if we wait a few more months we shall know. In the meantime, a few fillings carefully made in the mouths of persons who are willing to be used for experiment, will materially aid us in determining what use each of us individually may be able to make of the new method.

Considerable routine business was transacted, and the following members were elected to serve as officers for the ensuing year:

*President*—Benj. Cohen, Hamburg.

*Vice-President*—Wm. Patton, Cologne.

*Secretary*—F. Foerster, Berlin.

*Treasurer*—Wm. Sachs, Breslau.

The subject of improper graduation in America, which is now receiving so much attention in various parts of Europe, particularly in Germany, was discussed at some length, and the following resolutions were passed:

1. Candidates for actual membership of the American Dental Society of Europe will be required to make the Membership Committee acquainted with the name of the dental college where they graduated, and the length of time engaged in study at the same.

2. Any graduate of the Pennsylvania Dental College, who may apply for membership, shall be obliged to read before the society an essay on a scientific dental subject, in the English language, with satisfactory evidence that he is the author of the same.

